# Research on Government-Enterprise Regulation of Online Car-Hailing Based on Differential Game

**DOI:** 10.3389/fpsyg.2022.925028

**Published:** 2022-07-13

**Authors:** Mingge Yang, Yajie Liu, Lulu Sun, Danning Wang, Xiaozhen Liang

**Affiliations:** Department of Management Science and Engineering, School of Management, Shanghai University, Shanghai, China

**Keywords:** online car-hailing, regulatory effort, differential game, cost subsidy, benefit function

## Abstract

In the Internet era, with the widespread application of digital technology, the way people travel has changed. Compared with traditional taxis, more and more people prefer to choose online car-hailing. The rapid development of the online car-hailing industry has solved the problem of taxi-hailing to a certain extent, but it has also brought some new problems. To change the dilemma of the online car-hailing industry, it is necessary to strengthen the regulation of the online car-hailing industry. In this study, we consider the regulatory system composed of a local government and an enterprise and use the differential game to study the regulation of online car-hailing. In the Nash non-cooperative game, Stackelberg master–slave game, and cooperative game, we, respectively, investigate the indicators, such as the optimal regulatory effort of the government, the optimal regulatory effort of the enterprise, the optimal benefit function of the government, the optimal benefit function of the enterprise, the optimal benefit function of the system, the optimal trajectory of the service quality level for the enterprise, and the optimal trajectory of the goodwill for the enterprise. Moreover, we analyze the corresponding conclusions through examples. We obtained some important results. (i) In the Stackelberg master–slave game, the optimal ratio of the local government subsidy to the enterprise's regulatory cost is only related to the benefit distribution coefficient and has nothing to do with other factors. Moreover, when the benefit distribution coefficient is >1/3, the local government is willing to share the regulatory cost of the enterprise. Otherwise, the local government refuses to share the regulatory cost of the enterprise. (ii) Compared with the Nash non-cooperative game, the optimal regulatory effort of the local government remains unchanged in the Stackelberg master–slave game, but the optimal benefit of the local government increases. Moreover, when the benefit distribution coefficient is >1/3, both the optimal regulatory effort and the optimal benefit of the enterprise increase. (iii) Compared with the Stackelberg master–slave game, in the cooperative game, the optimal regulatory effort of both government and enterprise increases, and the system's optimal benefit also increases. (iv) From the Nash non-cooperative game to the Stackelberg master–slave game and then to the cooperative game when the benefit distribution coefficient is >1/3, the service quality level and goodwill of the enterprise all increase.

## Introduction

The rapid development of the Internet has had a huge impact on many aspects of society, such as economic development, environmental regulation and protection, energy consumption, people's health, and university students' academic achievement (Li et al., 2018; Ren et al., 2021; Wu et al., 2021a,b,c; Hao et al., 2022; Wang et al., 2022). With the advent of the era of the Internet, digital technology has been widely used in all fields of society, which has led to the emergence of many new industries. These new industries have changed the way people produce and live. Based on this background, online car-hailing represented by Didi began to come into people's vision, which has changed the traditional way people travel. In recent years, the rapid development of online car-hailing has provided an opportunity for solving the problem of taxi-hailing and has alleviated the problem to some extent (Zhang, 2018). Relying on Internet digital technology, the online car-hailing sharing economy has broken the barrier of asymmetric information between traditional taxis and passengers. It not only changed the way people travel but also provided sustainable solutions to economic, social, environmental, and technological problems. However, it is undeniable that the development of online car-hailing also faces various problems in the process of changing the traditional taxi industry, which makes consumers face potential threats to their personal and property safety (Zuo et al., 2019). Currently, the regulation of online car-hailing in China is mainly carried out by online car-hailing platforms and local government separately. There is a lack of connectivity between online car-hailing platforms and local government. To maintain a good online car-hailing market, the regulatory mode of online car-hailing has been constantly innovated and changed. A new regulatory mode, “the government regulates the platforms and the platforms regulate the vehicles,” is gradually being accepted. Therefore, it is of great practical significance for us to discuss the regulation of government-enterprise online car-hailing.

Watane et al. (2016) analyzed Uber's business model and deeply discussed its platform ecosystem. They found that the development of Uber's platform ecosystem depends mainly on the joint promotion of the government and taxis. Wang (2016) believed that the mode “the government regulates the platforms, and the platforms regulate the drivers and vehicles” should be used to change the chaos in the online car-hailing market and achieve the purpose of regulation on the basis of complying with administrative regulations. Zhao and Li (2019) proposed that a mixed regulatory mode should be established to realize the healthy development of online car-hailing by means of “cooperative regulation + self-regulation.” Cannon and Summers (2014) pointed out that car-hailing platforms can gain the trust of the government and avoid conflicts with regulators by changing their work mode and taking the initiative to explain their business to regulators and share data. Cohen and Sundararajan (2015) stated that in the Internet economy, the government should change its mind in time and work with platforms to regulate online car-hailing because platforms already have the good self-regulatory ability.

The literature (Cannon and Summers, 2014; Cohen and Sundararajan, 2015; Wang, 2016; Watane et al., 2016; Zhao and Li, 2019) studied the regulation of government-enterprise online car-hailing from static perspectives such as the construction of regulatory systems and the improvement of regulatory methods. Because the regulation of online car-hailing often spans multiple cycles, it is closer to reality to study the regulation of government-enterprise online car-hailing from dynamic perspectives. For example, Fu and Shi (2019) used an evolutionary game to study the factors that affect the behavior of online car-hailing platforms and the choice of regulatory strategies. Moreover, they analyzed the objective existence of the regulatory dilemma for online car-hailing. Fu and Shi (2020) established an evolutionary game model consisting of online car-hailing platforms and government regulators. Moreover, they studied the influence of media reports on the behavioral preferences and strategic choices of main participants in the online car-hailing industry. The literature (Lei et al., 2020a; Weng and Luo, 2021) used the evolutionary game to study the game behavior between online car-hailing platforms and online car-hailing drivers in the background of the implementation of the new policy. Moreover, they found the evolutionary stability strategy (ESS). Fu and Shi (2021) constructed an evolutionary game model consisting of insurance companies and online car-hailing platforms and analyzed the factors that affect the strategic choices of insurance companies and online car-hailing platforms. Sun et al. (2019) established an evolutionary game model consisting of the government and platform and discussed whether the online car-hailing platform needs strict regulation in the current Internet environment. Li and Zhang (2018) used a mixed Cournot duopoly dynamic game to analyze the relationship between the preference and executive power of the local government and the irresponsible governance of the enterprise. Moreover, they deeply studied the equilibrium solution of the mixed Cournot duopoly dynamic game. Lei et al. (2020b) constructed a tripartite evolutionary game model composed of online car-hailing platforms, drivers, and consumers and discussed the regulation of the online car-hailing market from the perspective of multi-stakeholders. Wang et al. (2020) built an evolutionary game model composed of the government, online car-hailing platforms, and drivers and analyzed the choice and stability of strategies for the government regulation, the platform regulation, and the driver operation. Li and Wang (2019) established a dynamic game model with incomplete information composed of the government, online car-hailing platforms, and consumers. Moreover, they discussed the regulation of online car-hailing with the participation of consumers.

The literature (Li and Zhang, 2018; Fu and Shi, 2019, 2020, 2021; Li and Wang, 2019; Sun et al., 2019; Lei et al., 2020a,b; Wang et al., 2020; Weng and Luo, 2021) all studied the regulation of government-enterprise online car-hailing from a dynamic perspective. They used the evolutionary game, mixed Cournot duopoly dynamic game, and sequence game. So far, few scholars have studied the regulation of online car-hailing by using the differential game. In fact, the differential game is one type of dynamic game that has been widely used in the fields of production and operations, quality control, and advertising promotion of supply chain enterprises (De Giovanni, 2011; Li, 2020; Yang et al., 2021, 2022b). Yang et al. (2022a) regarded the local government, online car-hailing platforms, and drivers as a regulatory system and established a stochastic differential game to analyze the impact of central government subsidy and alliance mechanism on the decision-making of the system members. It is worth mentioning that they discussed the regulation of online car-hailing from the perspective of the tripartite game. In practice, the local government and online car-hailing platforms are more involved in the regulation of online car-hailing, while online car-hailing drivers rarely participate in the regulation of online car-hailing. So the tripartite game established by Yang et al. (2022a) does not conform to reality. Compared with the tripartite game, the two-party game between the local government and online car-hailing platforms is more realistic. In addition, Yang et al. (2022a) not only considered the impact of central government subsidy on the decision-making of the system members but also regarded central government subsidy as an exogenous variable unaffected by the system. In practice, the local government usually shares the regulatory cost of the enterprise through tax incentives and financial subsidies, while the central government rarely shares the regulatory cost of the enterprise. Moreover, subsidy decisions of the central government often interact with the regulatory decisions of the enterprise and are influenced by the system. Furthermore, so far no scholars have considered the impact of the service quality level for the enterprise on the regulation of online car-hailing. In fact, the service quality level of the enterprise is dynamically changed with time and has an important impact on the regulatory benefit of online car-hailing. For example, Shenzhou Taxi has improved its service level and brand image to attract consumers by changing the old operation mechanism and regulatory mode.

Table 1 intuitively shows the contributions of this study and the differences between this study and the previous literature. (i) The expression for the optimal ratio of the local government subsidy to the enterprise's regulatory cost is given, whereas it was not given in previous literature. (ii) The impact of the enterprise service quality level on the regulation of online car-hailing is considered in this study, whereas it was not considered in previous literature.

**Table 1:** Summary of relevant literature.

**Reference**	**Model type**		**Dynamic game**			**Expression for the optimal ratio of the local government subsidy to the enterprise's regulatory cost**	**Goodwill**	**Service quality level**
	**Static**	**Dynamic**	**Evolutionary game**	**Sequence game**	**Differential game**			
Cannon and Summers (2014)	√							
Cohen and Sundararajan (2015)	√							
Wang (2016)	√							
Watane et al. (2016)	√							
Zhao and Li (2019)	√							
Fu and Shi (2019)		√	√					
Fu and Shi (2020)		√	√					
Fu and Shi (2021)		√	√					
Lei et al. (2020a)		√	√					
Lei et al. (2020b)		√	√					
Li and Wang (2019)		√	√	√				
Li and Zhang (2018)		√	√					
Sun et al. (2019)		√	√					
Wang et al. (2020)		√	√					
Weng and Luo (2021)		√	√					
Yang et al. (2022a)		√			√		√	
Present study		√			√	√	√	√

Inspired by the above research, we consider the impact of service quality level and goodwill for the enterprise on the regulatory benefit of online car-hailing. By using the differential game, we study some indicators in the Nash non-cooperative game, Stackelberg master–slave game, and cooperative game. These indicators are the optimal regulatory effort of the government, the optimal regulatory effort of the enterprise, the optimal benefit function of the government, the optimal benefit function of the enterprise, the optimal benefit function of the system, the optimal trajectory of service quality for the enterprise, and the optimal trajectory of goodwill for the enterprise. By comparing and analyzing the corresponding results of three game scenarios, we can provide some theoretical guidance for the regulation of government-enterprise online car-hailing.

## Model Assumptions

In this study, we consider the simple regulatory system, which is composed of a local government (G) and an enterprise (E). On this basis, we study the regulation of online car-hailing. The local government strengthens regulation of online car-hailing platforms through some means, such as strengthening administrative management and identifying barriers to entry. The enterprise regulates online car-hailing through some means, such as verifying the criminal background and driving records of online car-hailing drivers.

**Assumption 1:** Referring to the assumption of effort cost for quality control in Hong and Huang (2016), it is assumed that the regulatory cost is a convex function of the regulatory effort. Then, we can assume that the regulatory cost of the local government and the enterprise at the time t are


$$\begin{array}{l} C_{G}\left(t\right)=\frac{1}{2}K_{G}E_{G}^{2}\left(t\right),C_{E}\left(t\right)=\frac{1}{2}K_{E}E_{E}^{2}\left(t\right), \end{array}$$

respectively, where *C*_*G*_(*t*) and *C*_*E*_(*t*) represent the regulatory cost of the local government and the enterprise at time *t*. *K*_*G*_ and *K*_*E*_ represent the cost coefficients of regulatory effort for the local government and the enterprise. *K*_*G*_ > 0, *K*_*E*_ > 0. *E*_*G*_(*t*) and *E*_*E*_(*t*) represent the regulatory effort of the local government and the enterprise at the time *t*.

**Assumption 2:** The service quality level of the enterprise is a dynamic process and is jointly determined by the regulatory effort of the local government and the enterprise. On the one hand, the local government strengthens regulation of online car-hailing platforms through some means, such as strengthening administrative management and identifying barriers to entry. Under strong regulation of the government, the platform can effectively restrain the behavior of online car-hailing. Thus, the service quality level of the enterprise can be improved. On the other hand, the enterprise regulates online car-hailing through some means, such as verifying the criminal background and driving records of online car-hailing drivers. Thus, the life and property safety of passengers can be protected, and the service quality level of the enterprise can be improved. Referring to the assumption of the service quality level for the enterprise in Wu et al. (2017), we can assume that the changing rule of the service quality level of the enterprise with time is


(1) $$\begin{array}{l} {\dot{K}\left(t\right)=\mu_{G}E}_{G}\left(t\right)+{\mu_{E}E}_{E}\left(t\right)-\delta K\left(t\right), \end{array}$$

where *K*(*t*) represents the service quality level of the enterprise at time *t*, and the initial service quality level *K*(0) = *K*_0_ ≥ 0. μ_*G*_ and μ_*E*_, respectively, represent the influence coefficients of the regulatory effort of the local government and the enterprise on the service quality level of the enterprise. μ_*G*_ > 0, μ_*E*_ > 0. δ represents the attenuation coefficient of the service quality level for the enterprise.

**Assumption 3:** The goodwill of the enterprise is simultaneously positively affected by the regulatory effort and the service quality level of the enterprise. Considering the natural attenuation of the goodwill for the enterprise, we adopt the deformation of the goodwill model in Nerlove and Arrow (1962) to represent the changing rule of the enterprise goodwill over time, that is,


(2) $$\begin{array}{l} Ġ\left(t\right)=\alpha E_{E}\left(t\right)+\beta K\left(t\right)-\phi G\left(t\right), \end{array}$$

where *G*(*t*) represents the goodwill of the enterprise at the time *t*, and the initial goodwill *G*(0) = *G*_0_ ≥ 0. α and β represent the influence coefficients of the enterprise's regulatory effort and service quality level on the goodwill. α > 0, β > 0. ϕ represents the attenuation rate of the enterprise goodwill, which is usually caused by the launch of new products and consumers' forget, and ϕ > 0.

**Assumption 4:** The regulatory effort of the local government has an indirect impact on the benefit, while the regulatory effort, service quality level, and goodwill of the enterprise have a direct impact on the benefit. Therefore, it is assumed that the total benefit of the regulation for the government-enterprise online car-hailing is


$$\begin{array}{l} R\left(t\right)=\varphi +\gamma E_{E}\left(t\right)+\tau K\left(t\right)+\theta G\left(t\right), \end{array}$$

where φ represents the potential benefit when the enterprise does not invest effort in regulation, and φ > 0. γ represents the influence coefficient of the degree of regulatory effort of the enterprise on the benefit, and γ > 0. τ and θ, respectively, represent the influence coefficients of the service quality level and goodwill on the benefit. τ > 0, θ > 0.

**Assumption 5:** To encourage the enterprise to take the initiative to implement regulatory strategies, the local government as the leader of the regulatory system should subsidize the regulatory cost of the enterprise. The ratio of the local government subsidy to the enterprise's regulatory cost is *S*(*t*), and 0 ≤ *S*(*t*) < 1, which implies that the government only bears part of the regulatory cost of the enterprise.

**Assumption 6:** It is assumed that the total benefit of the regulation for the government-enterprise online car-hailing is shared by both parties. The local government gets ω and the enterprise gets 1 − ω, where ω is the benefit distribution coefficient. Moreover, ω is determined in advance by both parties and ω ∈ (0, 1).

**Assumption 7:** The local government and the enterprise have the same discount rate ρ > 0. They seek the optimal regulatory strategy to maximize their own benefit in an infinite time zone.

**Assumption 8:** Since the relevant parameters in the model are time-independent, then the government and enterprise are playing the same game at any time in an infinite period. The optimal strategies of the government and the enterprise are determined by a static feedback control strategy. For the convenience of writing, the time *t* will be omitted in the following text.

## Model Construction and Solution

### Nash Non-Cooperative Game

In the Nash non-cooperative game, the local government and the enterprise make decisions independently at the same time to maximize their own benefit. At this time, the local government will not provide any regulatory subsidy for the enterprise, that is, *S*(*t*) = 0. The Nash non-cooperative game is denoted by a superscript *N*, then the decision-making problems of the local government and the enterprise are


$$\begin{array}{l} \;\underset{{\text{E}}_{\text{G}}}{\text{max}}J_{G}^{N}=\int_{0}^{\infty }e^{-\rho t}\left[\omega \left(\varphi +\gamma E_{E}+\tau K+\theta G\right)-\frac{1}{2}K_{G}E_{G}^{2}\right]dt,\\ \underset{{\text{E}}_{\text{E}}}{\text{max}}J_{E}^{N}=\int_{0}^{\infty }e^{-\rho t}\left[\left(1-\omega \right)\left(\varphi +\gamma E_{E}+\tau K+\theta G\right)-{{\frac{1}{2}K}_{E}E}_{E}^{2}\right]dt. \end{array}$$

**Proposition 1:** In the Nash non-cooperative game, the optimal regulatory effort of the local government is


(3) $$\begin{array}{l} {\;E}_{G}^{N^{*}}=\frac{\omega \mu_{G}}{K_{G}\left(\rho +\delta \right)}\left(\frac{\theta \beta }{\phi +\rho }+\tau \right), \end{array}$$

and the optimal regulatory effort of the enterprise is


(4) $$\begin{array}{l} {\;E}_{E}^{N^{*}}=\frac{1-\omega }{K_{E}}\left[\gamma +\frac{\mu_{E}}{\rho +\delta }\left(\frac{\theta \beta }{\phi +\rho }+\tau \right)+\frac{\theta \alpha }{\phi +\rho }\right]. \end{array}$$

**Proof:** According to the optimal control theory, for any *K, G* ≥ 0, both $V_{G}^{N}\left(K,G\right)$ and $V_{E}^{N}\left(K,G\right)$ satisfy the Hamilton-Jacobi-Bellman (HJB) equation, that is,


(5) $$\begin{array}{r} \rho V_{G}^{N}\left(K,G\right)=\underset{E_{G}}{max}[\omega \left(\varphi +\gamma E_{E}+\tau K+\theta G\right)-\frac{1}{2}K_{G}E_{G}^{2}+V_{GK}^{N^{\prime }}\left({\mu_{G}E}_{G}+{\mu_{E}E}_{E}-\delta K\right)\\ \;{\;+V}_{GG}^{N^{\prime }}\left(\alpha E_{E}+\beta K-\phi G\right)], \end{array}$$

(6) $$\begin{array}{r} \;\rho V_{E}^{N}\left(K,G\right)=\underset{E_{E}}{max}[\left(1-\omega \right)\left(\varphi +\gamma E_{E}+\tau K+\theta G\right)-\frac{1}{2}{K_{E}E}_{E}^{2}\;+V_{EK}^{N^{\prime }}\left({\mu_{G}E}_{G}+{\mu_{E}E}_{E}-\delta K\right)\\ \;+V_{EG}^{N^{\prime }}\left(\alpha E_{E}+\beta K-\phi G\right)] \end{array}$$

Taking the first-order partial derivative of the function on the right-hand side of (5) with respect to *E*_*G*_ and setting the partial derivative equal to zero, we get


(7) $$\begin{array}{l} {\;E}_{G}=\frac{\mu_{G}V_{GK}^{N^{\prime }}}{K_{G}}. \end{array}$$

Taking the first-order partial derivative of the function on the right-hand side of (6) with respect to *E*_*E*_ and setting the partial derivative equal to zero, we get


(8) $$\begin{array}{l} {\;E}_{E}=\frac{\left(1-\omega \right)\gamma +\mu_{E}V_{EK}^{N^{\prime }}+\alpha V_{EG}^{N^{\prime }}}{K_{E}}. \end{array}$$

Substituting (7) and (8) into (5), we obtain


(9) $$\begin{array}{l} \rho V_{G}^{N}\left(K,G\right)=\left(\omega \tau -\delta V_{GK}^{N^{\prime }}+\beta V_{GG}^{N^{\prime }}\right)K+\left(\omega \theta -\phi V_{GG}^{N^{\prime }}\right)G\\ +\omega \varphi +\frac{\mu_{G}^{2}{\left(V_{GK}^{N^{\prime }}\right)}^{2}}{{2K}_{G}}\\ +\frac{\left[\left(1-\omega \right)\gamma +\mu_{E}V_{EK}^{N^{\prime }}+\alpha V_{EG}^{N^{\prime }}\right]\left(\omega \gamma +\mu_{E}V_{GK}^{N^{\prime }}+\alpha V_{GG}^{N^{\prime }}\right)}{K_{E}}, \end{array}$$

Substituting (7) and (8) into (6), we obtain


(10) $$\begin{array}{l} \rho V_{E}^{N}\left(K,G\right)=\left[\left(1-\omega \right)\tau -\delta V_{EK}^{N^{\prime }}+\beta V_{EG}^{N^{\prime }}\right]K\\ +\left[\left(1-\omega \right)\theta -\phi V_{EG}^{N^{\prime }}\right]G+\left(1-\omega \right)\varphi \\ +\frac{{\mu_{G}^{2}V_{EK}^{N^{\prime }}V}_{GK}^{N^{\prime }}}{K_{G}}+\frac{{\left[\left(1-\omega \right)\gamma +\mu_{E}V_{EK}^{N^{\prime }}+\alpha V_{EG}^{N^{\prime }}\right]}^{2}}{{2K}_{E}}. \end{array}$$

According to the structures of (9) and (10), it is assumed that the linear analytical expressions of the optimal benefit functions $V_{G}^{N}\left(K,G\right)$ and $V_{E}^{N}\left(K,G\right)\;$about *K* and *G* are, respectively, as follows:


(11) $$\begin{array}{l} {\;V}_{G}^{N}\left(K,G\right)=m_{1}K+m_{2}G+m_{3}, \end{array}$$

(12) $$\begin{array}{l} {\;V}_{E}^{N}\left(K,G\right)=r_{1}K+r_{2}G+r_{3}, \end{array}$$

where *m*_1_, *m*_2_, *m*_3_ and *r*_1_, *r*_2_, *r*_3_ are parameters to be determined. Substituting (11) and (12) into (9), we obtain the following results through the method of undetermined coefficients


(13) $$\begin{array}{l} {\;m}_{1}^{*}=\frac{\omega }{\rho +\delta }\left(\frac{\theta \beta }{\phi +\rho }+\tau \right), \end{array}$$

(14) $$\begin{array}{l} m_{2}^{*}=\frac{\omega \theta }{\phi +\rho }, \end{array}$$

(15) $$\begin{array}{l} m_{3}=\frac{\omega \phi }{\rho }+\frac{\mu_{G}^{2}{\left(m_{1}^{*}\right)}^{2}}{{2\rho K}_{G}}+\frac{\left[\left(1-\omega \right)\gamma +\mu_{E}r_{1}^{*}+\alpha r_{2}^{*}\right]\left(\omega \gamma +\mu_{E}m_{1}^{*}+\alpha m_{2}^{*}\right)}{\rho K_{E}}. \end{array}$$

Substituting (11) and (12) into (10), we obtain the following results through the method of undetermined coefficients


(16) $$\begin{array}{l} r_{1}^{*}=\frac{1-\omega }{\rho +\delta }\left(\frac{\theta \beta }{\phi +\rho }+\tau \right), \end{array}$$

(17) $$\begin{array}{l} r_{2}^{*}=\frac{\left(1-\omega \right)\theta }{\phi +\rho }, \end{array}$$

(18) $$\begin{array}{l} {\;r}_{3}=\frac{\left(1-\omega \right)\varphi }{\rho }+\frac{\mu_{G}^{2}r_{1}^{*}m_{1}^{*}}{\rho K_{G}}+\frac{{\left[\left(1-\omega \right)\gamma +\mu_{E}r_{1}^{*}+\alpha r_{2}^{*}\right]}^{2}}{{2\rho K}_{E}}. \end{array}$$

Substituting (13) into (7), we obtain the optimal regulatory effort of the local government as shown in (3). Substituting (16) and (17) into (8), we obtain the optimal regulatory effort of the enterprise as shown in (4).      **Q.E.D**.

**Proposition 2:** In the Nash non-cooperative game, the optimal benefit function of the local government is


(19) $$\begin{array}{l} V_{G}^{N^{*}}\left(K,G\right)=\frac{\omega }{\rho +\delta }\left(\frac{\theta \beta }{\phi +\rho }+\tau \right)K+\frac{\omega \theta }{\phi +\rho }G+\frac{\omega \varphi }{\rho }\\ \;+\frac{\omega^{2}\mu_{G}^{2}}{2\rho K_{G}{\left(\rho +\delta \right)}^{2}}{\left(\frac{\theta \beta }{\phi +\rho }+\tau \right)}^{2}+\frac{{\left(1-\omega \right)\omega M}^{2}}{\rho K_{E}}, \end{array}$$

the optimal benefit function of the enterprise is


(20) $$\begin{array}{l} \;V_{E}^{N^{*}}\left(K,G\right)=\frac{1-\omega }{\rho +\delta }\left(\frac{\theta \beta }{\phi +\rho }+\tau \right)K+\frac{\left(1-\omega \right)\theta }{\phi +\rho }G+\\ \frac{\left(1-\omega \right)\varphi }{\rho }+\frac{\left(1-\omega \right)\omega \mu_{G}^{2}}{\rho K_{G}{\left(\rho +\delta \right)}^{2}}{\left(\frac{\theta \beta }{\phi +\rho }+\tau \right)}^{2}+\frac{{{\left(\omega -1\right)}^{2}M}^{2}}{{2\rho K}_{E}} \end{array}$$

and the optimal benefit function of the system is


(21) $$\begin{array}{r} V_{S}^{N^{*}}\left(K,G\right)=\frac{1}{\rho +\delta }\left(\frac{\theta \beta }{\phi +\rho }+\tau \right)K+\frac{\theta }{\phi +\rho }G+\frac{\varphi }{\rho }+\\ \frac{\left(2-\omega \right)\omega \mu_{G}^{2}}{{2\rho K}_{G}{\left(\rho +\delta \right)}^{2}}{\left(\frac{\theta \beta }{\phi +\rho }+\tau \right)}^{2}+\frac{{\left(1-\omega^{2}\right)M}^{2}}{{2\rho K}_{E}}, \end{array}$$

where $M=\gamma +\frac{\mu_{E}}{\rho +\delta }\left(\frac{\theta \beta }{\phi +\rho }+\tau \right)+\frac{\theta \alpha }{\phi +\;\rho }$.

**Proof:** Substituting (13), (14), (16), and (17) into (15), we obtain


(22) $$\begin{array}{r} m_{3}^{*}=\frac{\omega \varphi }{\rho }+\frac{\omega^{2}\mu_{G}^{2}}{2\rho K_{G}{\left(\rho +\delta \right)}^{2}}{\left(\frac{\theta \beta }{\phi +\rho }+\tau \right)}^{2}\\ +\frac{{\left(1-\omega \right)\omega M}^{2}}{\rho K_{E}}. \end{array}$$

Substituting (13), (16), and (17) into (18), we obtain


(23) $$\begin{array}{l} r_{3}^{*}=\frac{\left(1-\omega \right)\varphi }{\rho }+\frac{\left(1-\omega \right)\omega \mu_{G}^{2}}{\rho K_{G}{\left(\rho +\delta \right)}^{2}}{\left(\frac{\theta \beta }{\phi +\rho }+\tau \right)}^{2}\\ +\frac{{{\left(\omega -1\right)}^{2}M}^{2}}{{2\rho K}_{E}}. \end{array}$$

Substituting (13), (14), and (22) into (11), we obtain the optimal benefit function of the local government as shown in (19). Substituting (16), (17), and (23) into (12), we obtain the optimal benefit function of the enterprise as shown in (20). Furthermore, we obtain the optimal benefit function of the system as shown in (21).      **Q.E.D**.

**Proposition 3:** In the Nash non-cooperative game, the optimal trajectory of the service quality level for the enterprise is


(24) $$\begin{array}{l} K^{N^{*}}\left(t\right)=\left(K_{0}-K_{\infty }^{N}\right)e^{-\delta t}+K_{\infty }^{N}, \end{array}$$

and the optimal trajectory of the goodwill for the enterprise is


(25) $$\begin{array}{l} {\;G}^{N^{*}}\left(t\right)=G_{\infty }^{N}+\frac{\beta }{\phi -\delta }\left(K_{0}-K_{\infty }^{N}\right)e^{-\delta t}+\left[G_{0}-G_{\infty }^{N}-\frac{\beta }{\phi -\delta }\left(K_{0}-K_{\infty }^{N}\right)\right]e^{-\phi t}, \end{array}$$

where


$$\begin{array}{r} K_{\infty }^{N}=\frac{\omega \mu_{G}^{2}}{\delta K_{G}\left(\rho +\delta \right)}\left(\frac{\theta \beta }{\phi +\rho }+\tau \right)+\frac{\left(1-\omega \right)\mu_{E}}{\delta K_{E}}\left[\gamma +\frac{\mu_{E}}{\rho +\delta }\left(\frac{\theta \beta }{\phi +\rho }+\tau \right)+\frac{\theta \alpha }{\phi +\rho }\;\right],\\ {\;G}_{\infty }^{N}=\frac{1-\omega }{\phi K_{E}}\left(\alpha +\frac{\beta \mu_{E}}{\delta }\right)\left[\gamma +\frac{\mu_{E}}{\rho +\delta }\left(\frac{\theta \beta }{\phi +\rho }+\tau \right)+\frac{\theta \alpha }{\phi +\rho }\right]+\frac{\omega \beta \mu_{G}^{2}}{\phi \delta K_{G}\left(\rho +\delta \right)}\left(\frac{\theta \beta }{\phi +\rho }+\tau \;\right). \end{array}$$

**Proof:** Substituting (3) and (4) into (1), we get


(26) $$\begin{array}{r} \dot{K}\left(t\right)=\frac{\omega \mu_{G}^{2}}{K_{G}\left(\rho +\delta \right)}\left(\frac{\theta \beta }{\phi +\rho }+\tau \right)+\frac{{\left(1-\omega \right)\mu }_{E}}{K_{E}}\left[\gamma +\frac{\mu_{E}}{\rho +\delta }\left(\frac{\theta \beta }{\phi +\rho }+\tau \right)+\frac{\theta \alpha }{\phi +\rho }\right]\\ -\delta K\left(t\right). \end{array}$$

Substituting (4) and (24) into (2), we get


(27) $$\begin{array}{r} Ġ\left(t\right)=\frac{\left(1-\omega \right)\alpha }{K_{E}}\left[\gamma +\frac{\mu_{E}}{\rho +\delta }\left(\frac{\theta \beta }{\phi +\rho }+\tau \right)+\frac{\theta \alpha }{\phi +\rho }\right]\\ +\beta \left[\left(K_{0}-K_{\infty }^{N}\right)e^{-\delta t}+K_{\infty }^{N}\right]-\phi G\left(t\right). \end{array}$$

By solving the differential equation (26), we can obtain the optimal trajectory of the service quality level for the enterprise as shown in (24). By solving the differential equation (27), we can obtain the optimal trajectory of the goodwill for the enterprise as shown in (25).      **Q.E.D**.

### Stackelberg Master–Slave Game

In the Stackelberg master–slave game, the local government is the leader of the government-enterprise regulation of online car-hailing, and the enterprise is the follower of the government-enterprise regulation of online car-hailing. To stimulate the enterprise to invest more in regulation, the local government is willing to share some of the regulatory costs of the enterprise. The decision-making process is as follows: First, the local government decides its own regulatory effort and the ratio *S*(*t*). Second, the enterprise decides its own regulatory effort according to the regulatory effort of the local government. From a long-term dynamic perspective, the local government and the enterprise form the Stackelberg non-cooperative game, which is denoted by superscript *D*. At this time, the decision-making problems of the local government and enterprise are


$$\begin{array}{l} \underset{{\text{E}}_{\text{G}},\text{S}}{\text{max}}J_{G}^{D}=\int_{0}^{\infty }e^{-\rho t}\left[\omega \left(\varphi +\gamma E_{E}+\tau K+\theta G\right)-\frac{1}{2}{K_{G}E}_{G}^{2}-\;\frac{1}{2}{SK_{E}E}_{E}^{2}\right]dt,\\ \;\underset{{\text{E}}_{\text{E}}}{\text{max}}J_{E}^{D}=\int_{0}^{\infty }e^{-\rho t}\left[\left(1-\omega \right)\left(\varphi +\gamma E_{E}+\tau K+\theta G\right)-\frac{1}{2}\left(1-S\right)K_{E}E_{E}^{2}\right]dt. \end{array}$$

**Proposition 4:** In the Stackelberg master–slave game, the optimal regulatory effort of the local government is


$$\begin{array}{l} {\;E}_{G}^{D^{*}}=\frac{\omega \mu_{G}}{K_{G}\left(\rho +\delta \right)}\left(\frac{\theta \beta }{\phi +\rho }+\tau \right), \end{array}$$

the optimal ratio of the local government subsidy to the enterprise's regulatory cost is


$$\begin{array}{l} S^{*}=\{\begin{array}{c} \frac{3\omega -1}{1+\omega },\;if\;\omega >\frac{1}{3},\\ 0,\;if\;\omega \leq \frac{1}{3}.\; \end{array} \end{array}$$

and the optimal regulatory effort of the enterprise is


$$\begin{array}{l} E_{E}^{D^{*}}=\frac{1+\omega }{2K_{E}}\left[\gamma +\frac{\mu_{E}}{\rho +\delta }\left(\frac{\theta \beta }{\phi +\rho }+\tau \right)+\frac{\theta \alpha }{\phi +\rho }\right]. \end{array}$$

**Proposition 5:** In the Stackelberg master–slave game, the optimal benefit function of the local government is


$$\begin{array}{r} {\;V}_{G}^{D^{*}}\left(K,G\right)=\frac{\omega }{\rho +\delta }\left(\frac{\theta \beta }{\phi +\rho }+\tau \right)K+\frac{\omega \theta }{\phi +\rho }G+\frac{\omega \varphi }{\rho }\\ +\frac{\omega^{2}\mu_{G}^{2}}{2\rho K_{G}{\left(\rho +\delta \right)}^{2}}{\left(\frac{\theta \beta }{\phi +\rho }+\tau \right)}^{2}+\frac{{{\left(\omega +1\right)}^{2}M}^{2}}{8\rho K_{E}}, \end{array}$$

the optimal benefit function of the enterprise is


$$\begin{array}{r} V_{E}^{D^{*}}\left(K,G\right)=\frac{1-\omega }{\rho +\delta }\left(\frac{\theta \beta }{\phi +\rho }+\tau \right)K+\frac{\left(1-\omega \right)\theta }{\phi +\rho }G+\frac{\left(1-\omega \right)\varphi }{\rho }\\ +\frac{\left(1-\omega \right)\omega \mu_{G}^{2}}{\rho K_{G}{\left(\rho +\delta \right)}^{2}}{\left(\frac{\theta \beta }{\phi +\rho }+\tau \right)}^{2}+\frac{{\left(1-\omega^{2}\right)M}^{2}}{{4\rho K}_{E}}, \end{array}$$

and the optimal benefit function of the system is


(28) $$\begin{array}{r} V_{S}^{D^{*}}\left(K,G\right)=\frac{1}{\rho +\delta }\left(\frac{\theta \beta }{\phi +\rho }+\tau \right)K+\frac{\theta }{\phi +\rho }G+\frac{\varphi }{\rho }\\ +\frac{\left(2-\omega \right)\omega \mu_{G}^{2}}{{2\rho K}_{G}{\left(\rho +\delta \right)}^{2}}{\left(\frac{\theta \beta }{\phi +\rho }+\tau \right)}^{2}+\frac{{\left(3-\omega \right)\left(1+\omega \right)M}^{2}}{{8\rho K}_{E}}, \end{array}$$

where $M=\gamma +\frac{\mu_{E}}{\rho +\delta }\left(\frac{\theta \beta }{\phi +\rho }+\tau \right)+\frac{\theta \alpha }{\phi +\rho }$.

Proposition 4 and Proposition 5 can be proved through the method of backward induction. First, we take the first-order partial derivative of the optimal benefit function $V_{E}^{D}\left(K,G\right)$ for the enterprise with respect to *E*_*E*_. Second, we take the first-order partial derivative of the optimal benefit function ${\;V}_{G}^{D}\left(K,G\right)$ for the local government with respect to *E*_*G*_ and *S*, respectively. The proof of Proposition 4 (Proposition 5) is similar to that of Proposition 1 (Proposition 2), so it is omitted here.

**Proposition 6:** In the Stackelberg master–slave game, the optimal trajectory of the service quality level for the enterprise is


(29) $$\begin{array}{l} {\;K}^{D^{*}}\left(t\right)=\left(K_{0}-K_{\infty }^{D}\right)e^{-\delta t}+K_{\infty }^{D}, \end{array}$$

and the optimal trajectory of the goodwill for the enterprise is


(30) $$\begin{array}{l} {\;G}^{D^{*}}\left(t\right)=G_{\infty }^{D}+\frac{\beta }{\phi -\delta }\left(K_{0}-K_{\infty }^{D}\right)e^{-\delta t}+\left[G_{0}-G_{\infty }^{D}-\frac{\beta }{\phi -\delta }\left(K_{0}-K_{\infty }^{D}\right)\right]e^{-\phi t} \end{array}$$

where


$$\begin{array}{r} K_{\infty }^{D}=\frac{\omega \mu_{G}^{2}}{\delta K_{G}\left(\rho +\delta \right)}\left(\frac{\theta \beta }{\phi +\rho }+\tau \right)+\frac{\left(1+\omega \right)\mu_{E}}{2\delta K_{E}}\left[\gamma +\frac{\mu_{E}}{\rho +\delta }\left(\frac{\theta \beta }{\phi +\rho }+\tau \right)+\frac{\theta \alpha }{\phi +\rho }\;\right],\\ G_{\infty }^{D}=\frac{1+\omega }{2\phi K_{E}}\left(\alpha +\frac{\beta \mu_{E}}{\delta }\right)\left[\gamma +\frac{\mu_{E}}{\rho +\delta }\left(\frac{\theta \beta }{\phi +\rho }+\tau \right)+\frac{\theta \alpha }{\phi +\rho }\right]+\frac{\omega \beta \mu_{G}^{2}}{\phi \delta K_{G}\left(\rho +\delta \right)}\left(\frac{\theta \beta }{\phi +\rho }+\tau \right). \end{array}$$

The proof of Proposition 6 is similar to that of Proposition 3, so it is omitted here.

### Cooperative Game

In the cooperative game, the local government and the enterprise make joint decisions to maximize the system benefit. The cooperative game is denoted by superscript *C*. At this time, the decision problem of the system is


$$\begin{array}{l} \underset{{\text{E}}_{\text{G}},{\text{E}}_{\text{E}}}{\text{max}}J_{S}^{C}=\int_{0}^{\infty }e^{-\rho t}\left[\left(\varphi +\gamma E_{E}+\tau K+\theta G\right)-\frac{1}{2}{K_{G}E}_{G}^{2}-\frac{1}{2}{K_{E}E}_{E}^{2}\right]dt. \end{array}$$

**Proposition 7:** In the cooperative game, the optimal regulatory effort of the local government is


$$\begin{array}{l} E_{G}^{C^{*}}=\frac{\mu_{G}}{K_{G}\left(\rho +\delta \right)}\left(\frac{\theta \beta }{\phi +\rho }+\tau \;\right), \end{array}$$

and the optimal regulatory effort of the enterprise is


$$\begin{array}{l} E_{E}^{C^{*}}=\frac{1}{K_{E}}\left[\gamma +\frac{\mu_{E}}{\rho +\delta }\left(\frac{\theta \beta }{\phi +\rho }+\tau \right)+\frac{\theta \alpha }{\phi +\rho }\;\right]. \end{array}$$

**Proposition 8:** In the cooperative game, the optimal benefit function of the system is


(31) $$\begin{array}{r} V_{S}^{C^{*}}\left(K,G\right)=\frac{1}{\rho +\delta }\left(\frac{\theta \beta }{\phi +\rho }+\tau \right)K+\frac{\theta }{\phi +\rho }G+\frac{\varphi }{\rho }\\ +\frac{\mu_{G}^{2}}{2\rho K_{G}{\left(\rho +\delta \right)}^{2}}{\left(\frac{\theta \beta }{\phi +\rho }+\tau \right)}^{2}+\frac{M^{2}}{{2\rho K}_{E}}, \end{array}$$

where $M=\gamma +\frac{\mu_{E}}{\rho +\delta }\left(\frac{\theta \beta }{\phi +\rho }+\tau \right)+\frac{\theta \alpha }{\phi +\rho }$.

**Proposition 9:** In the cooperative game, the optimal trajectory of the service quality level for the enterprise is


(32) $$\begin{array}{l} K^{C^{*}}\left(t\right)=\left(K_{0}-K_{\infty }^{C}\right)e^{-\delta t}+K_{\infty }^{C}, \end{array}$$

and the optimal trajectory of the goodwill for the enterprise is


(33) $$\begin{array}{l} G^{C^{*}}\left(t\right)=G_{\infty }^{C}+\frac{\beta }{\phi -\delta }\left(K_{0}-K_{\infty }^{C}\right)e^{-\delta t}+\left[G_{0}-G_{\infty }^{C}-\frac{\beta }{\phi -\delta }\left(K_{0}-K_{\infty }^{C}\right)\right]e^{-\phi t}, \end{array}$$

where


$$\begin{array}{r} K_{\infty }^{C}=\frac{\mu_{G}^{2}}{\delta K_{G}\left(\rho +\delta \right)}\left(\frac{\theta \beta }{\phi +\rho }+\tau \right)+\frac{\mu_{E}}{\delta K_{E}}\left[\gamma +\frac{\mu_{E}}{\rho +\delta }\left(\frac{\theta \beta }{\phi +\rho }+\tau \right)+\frac{\theta \alpha }{\phi +\rho }\;\right],\\ G_{\infty }^{C}=\frac{1}{\phi K_{E}}\left(\alpha +\frac{\beta \mu_{E}}{\delta }\right)\left[\gamma +\frac{\mu_{E}}{\rho +\delta }\left(\frac{\theta \beta }{\phi +\rho }+\tau \right)+\frac{\theta \alpha }{\phi +\rho }\right]+\frac{\beta \mu_{G}^{2}\;}{\varphi \delta K_{G}\left(\rho +\delta \right)}\left(\frac{\theta \beta }{\phi +\rho }+\tau \;\right). \end{array}$$

The proof of Proposition 7 (Proposition 8 and Proposition 9) is similar to that of Proposition 1 (Proposition 2 and Proposition 3), so it is omitted here.

## Comparison and Analysis

In the “Nash Non-cooperative Game” section, we obtain the optimal regulatory effort of the local government, the optimal regulatory effort of the enterprise, the optimal trajectory of the service quality level for the enterprise, the optimal trajectory of the goodwill for the enterprise, the optimal benefit function of the government, the optimal benefit function of the enterprise, and the optimal benefit function of the system under different game scenarios. In this section, we compare these results and draw some important conclusions.

**Corollary 1:** (1) The optimal regulatory effort of the local government satisfies $E_{G}^{N^{*}}{=E}_{G}^{D^{*}}<E_{G}^{C^{*}}$; (2) The optimal regulatory effort of the enterprise satisfies $E_{E}^{D^{*}}<E_{E}^{C^{*}}$. Especially, when $\omega >\frac{1}{3}$, we have ${E_{E}^{N^{*}}<E}_{E}^{D^{*}}$, and therefore ${E_{E}^{N^{*}}<E}_{E}^{D^{*}}<E_{E}^{C^{*}}$.

Since the regulatory effort of the local government has nothing to do with whether it provides the regulatory cost subsidy to the enterprise, we know that the optimal regulatory effort of the local government remains unchanged in the Stackelberg master–slave game compared with the Nash non-cooperative game. In the cooperative game, the regulatory activities of the local government and the enterprise as a whole are complementary. So the optimal regulatory effort of both local government and enterprise increases in the cooperative game compared with the Stackelberg master–slave game.

When the benefit distribution coefficient is $>\frac{1}{3}$, the optimal regulatory effort of the enterprise increases in the Stackelberg master–slave game compared with the Nash non-cooperative game. In fact, only when the benefit distribution coefficient is $>\frac{1}{3}$, the local government is willing to share the regulatory cost of the enterprise. Hence, the regulatory pressure on the enterprise is reduced, and the enthusiasm of the enterprise to invest in regulation is improved. It follows that the optimal regulatory effort of the enterprise increases.

**Corollary 2:** (1) When $\omega >\frac{1}{3}$, the optimal trajectory of the service quality level for the enterprise satisfies *K*^N^^*^(*t*) < *K*^D^^*^(*t*) < *K*^C^^*^(*t*); (2) When $\omega >\frac{1}{3}$, the optimal trajectory of the goodwill for the enterprise satisfies *G*^N^^*^(*t*) < *G*^D^^*^(*t*) < *G*^C^^*^(*t*).

According to Corollary 2, we know that when the benefit distribution coefficient is $>\frac{1}{3}$, from the Nash non-cooperative game to the Stackelberg master–slave game and then to the cooperative game, both the service quality level and goodwill of the enterprise are improved at the same moment. On the one hand, the regulatory effort of the local government and the enterprise has a positive impact on the service quality level of the enterprise, so the optimal regulatory effort of the enterprise increases in the Stackelberg master–slave game compared with the Nash non-cooperative game. It follows that the service quality level of the enterprise is improved. On the other hand, both the service quality level and regulatory effort of the enterprise positively affect the goodwill of the enterprise, so the goodwill of the enterprise is improved. That is, from the Nash non-cooperative game to the Stackelberg master–slave game and then to the cooperative game, the goodwill of the enterprise is improved at the same moment.

**Corollary 3:** (1) The optimal benefit function of the local government satisfies $V_{G}^{N^{*}}\left(K,G\right)<{\;V}_{G}^{D^{*}}\left(K,G\right)$; (2) when $\omega >\frac{1}{3}$, the optimal benefit function of the enterprise satisfies $V_{E}^{N^{*}}\left(K,G\right)<{\;V}_{E}^{D^{*}}\left(K,G\right)$.

Compared with the Nash non-cooperative game, in the Stackelberg master–slave game, the optimal benefit of the local government increases. Furthermore, when the benefit distribution coefficient is $>\frac{1}{3}$, the optimal benefit of the enterprise also increases. In fact, when the benefit distribution coefficient is $>\frac{1}{3}$, the optimal regulatory effort of the enterprise has a positive impact on the service quality level and goodwill of the enterprise and the system benefit. So the optimal benefit of the local government increases. When the benefit distribution coefficient is $>\frac{1}{3}$, the local government subsidy of regulatory cost for the enterprise can improve the enthusiasm of the enterprise to invest in regulation. So the optimal benefit of the enterprise increases.

**Corollary 4:** When $\omega >\frac{1}{3}$, the optimal benefit function of the system satisfies $V_{S}^{N^{*}}\left(K,G\right){<V}_{S}^{D^{*}}\left(K,G\right)<V_{S}^{C^{*}}\left(K,G\right)$.

Compared with the Nash non-cooperative game, in the Stackelberg master–slave game, when the benefit distribution coefficient is $>\frac{1}{3}$, the optimal benefit of both local government and enterprise increases. So the system benefit increases. Compared with the Stackelberg master–slave game, when the profit benefit coefficient is $>\frac{1}{3}$, the system benefit increases in the cooperative game. In fact, in the cooperative game, both the local government and enterprises make decisions to maximize the system benefit. So the optimal regulatory effort of both parties increases. It follows that the system benefit increases.

## Numerical Simulations

In this section, MatlabR2018a is used to carry out a numerical simulation on Corollary 2 and Corollary 4. In different game scenarios, the service quality level of the enterprise, the goodwill of the enterprise, and the system benefit all depends on the setting of model parameters. The benchmark parameters are set as follows:

μ_*G*_ = 0.8, μ_*E*_ = 0.8, δ = 0.2, α = 1, β = 0.5, ϕ = 0.3, φ = 2, γ = 1, τ = 0.5, θ = 0.5, *K*_*G*_ = 1, *K*_*E*_ = 1, ρ = 0.3, *K*_0_ = 0, *G*_0_ = 0.

**Figure 1:**
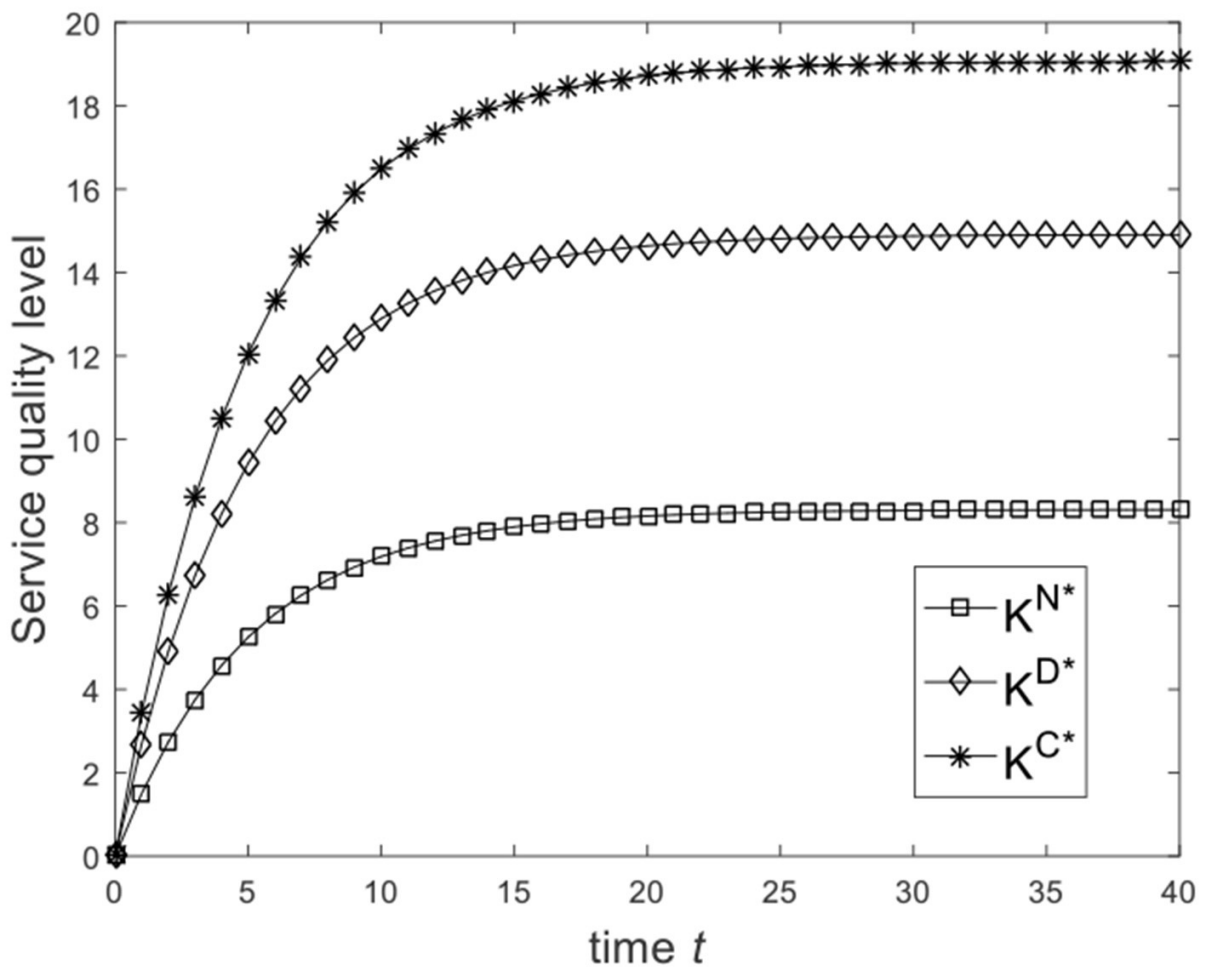
Diagram of enterprise service quality level for $\omega =\frac{2}{3}$.

**Figure 2:**
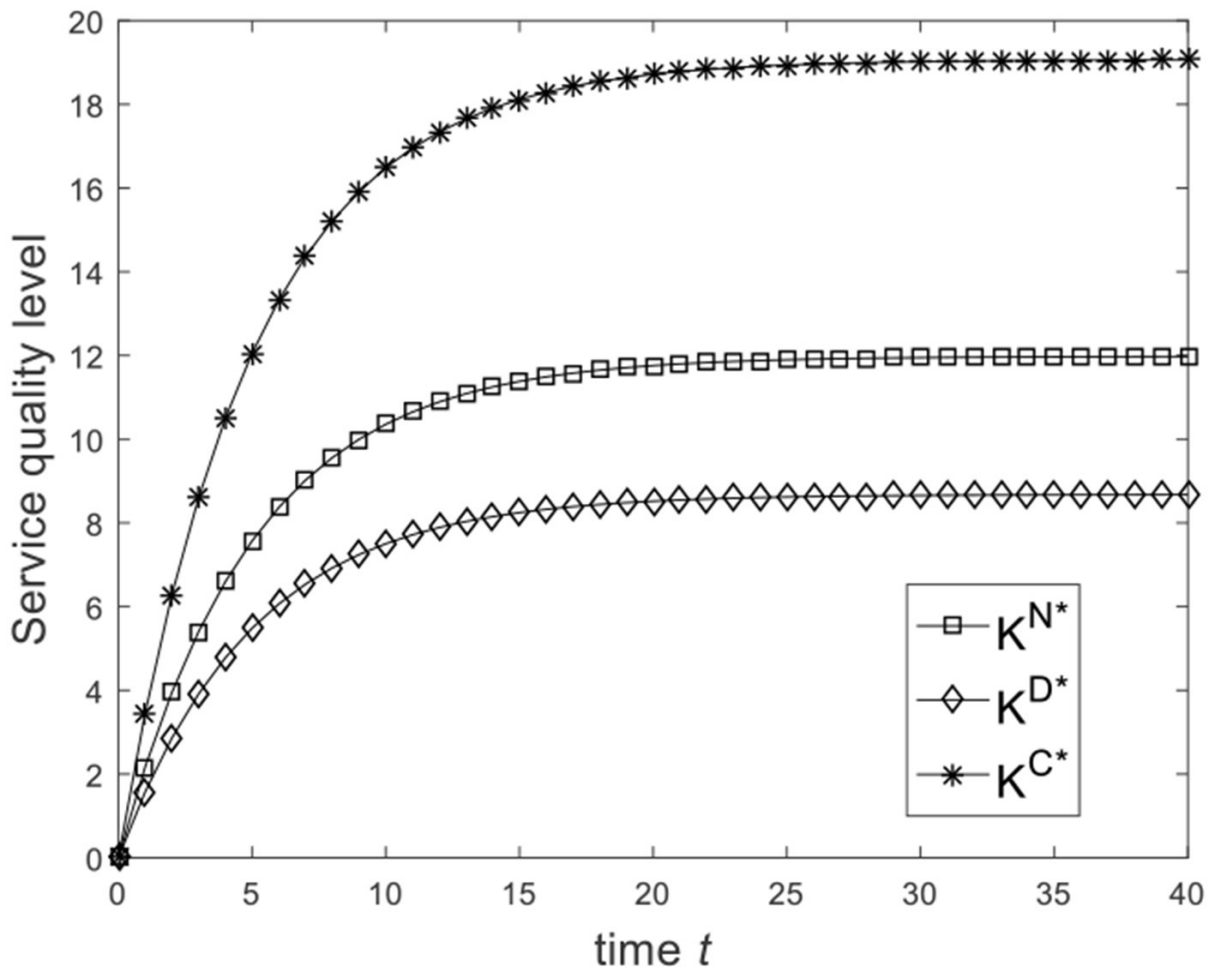
Diagram of enterprise service quality level for $\omega =\frac{1}{6}$.

Let $\omega =\frac{2}{3}$, and the benchmark parameters remain unchanged. Substituting benchmark parameters into (24), (29), and (32), we can get the service quality level of the enterprise under different game scenarios, as shown in Figure 1. From Figure 1, we know that from the Nash non-cooperative game to the Stackelberg master–slave game and then to the cooperative game, the service quality level of the enterprise at the same moment is improved. So Figure 1 is consistent with the conclusion of Corollary 2(1). Let $\omega =\frac{1}{6}$ and the benchmark parameters remain unchanged. Substituting benchmark parameters into (24), (29), and (32), we can get the service quality level of the enterprise under different game scenarios, as shown in Figure 2. From Figure 2, we know that the service quality level of the enterprise in the Stackelberg master–slave game is lower than that in the Nash non-cooperative game. So the condition $\omega >\frac{1}{3}$ is necessary for Corollary 2(1).

**Figure 3:**
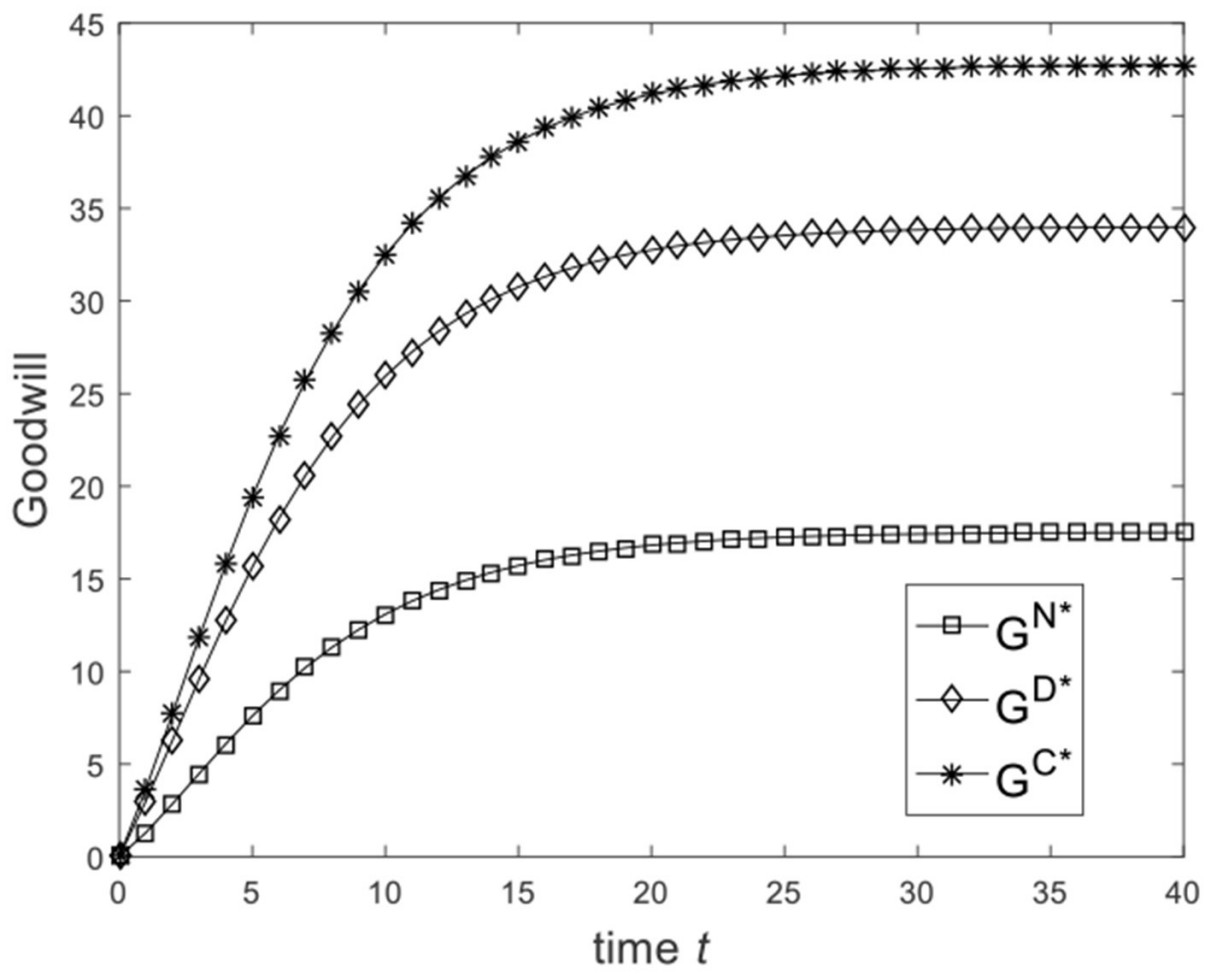
Diagram of enterprise goodwill for $\omega =\frac{2}{3}$.

**Figure 4:**
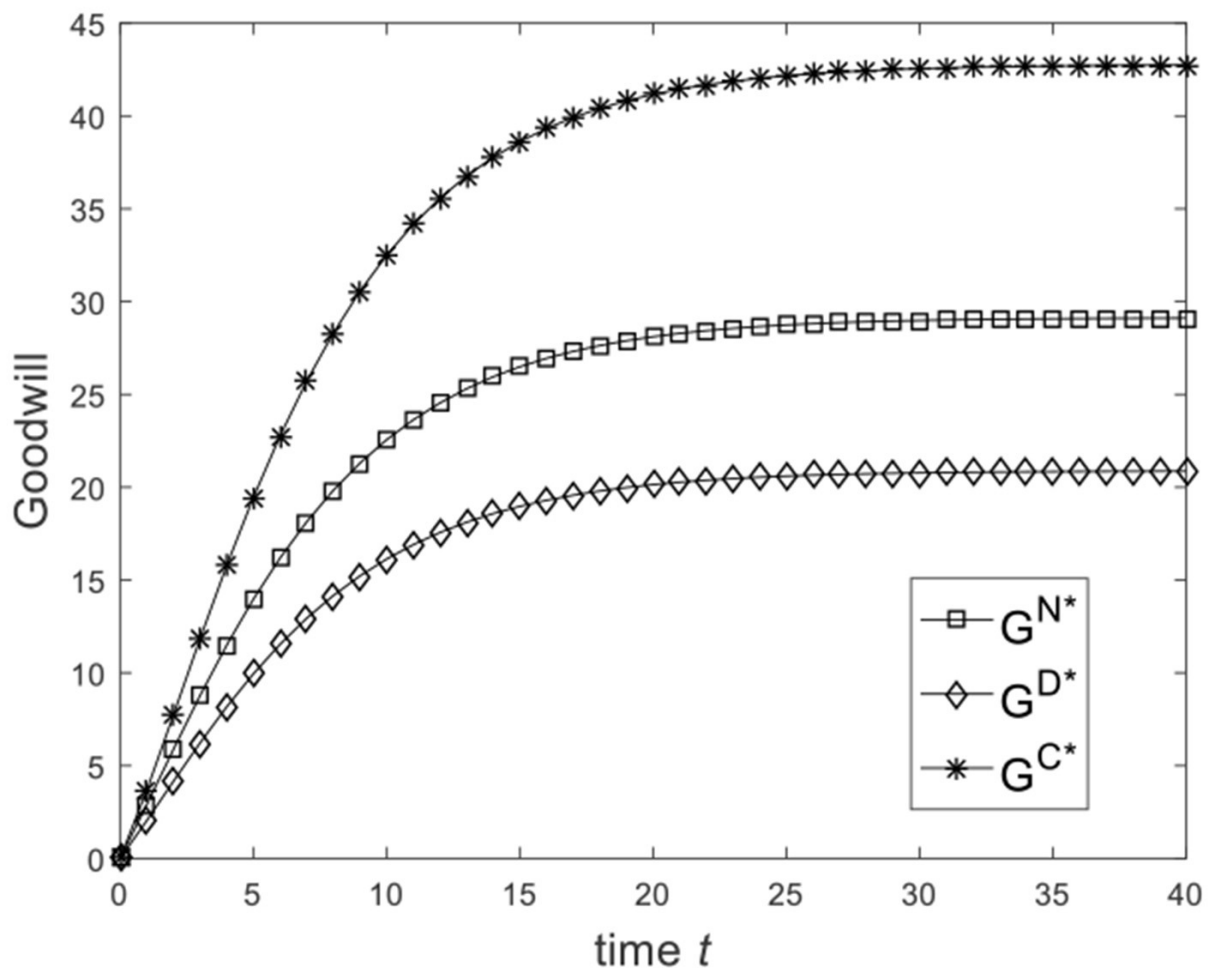
Diagram of enterprise goodwill for $\omega =\frac{1}{6}$.

Let $\omega =\frac{2}{3}$, and the benchmark parameters remain unchanged. Substituting benchmark parameters into (25), (30), and (33), we can obtain the goodwill of the enterprise under different game scenarios, as shown in Figure 3. From Figure 3, we know that from the Nash non-cooperative game to the Stackelberg master–slave game and then to the cooperative game, the goodwill of the enterprise at the same moment is improved. Figure 3 is consistent with the conclusion of Corollary 2(2). Let $\omega =\frac{1}{6}$, and the benchmark parameters remain unchanged. Substituting benchmark parameters into (25), (30), and (33), we can obtain the goodwill of the enterprise under different game scenarios, as shown in Figure 4. From Figure 4, we know that the goodwill of the enterprise in the Stackelberg master–slave game is lower than that in the Nash non-cooperative game. So the condition $\omega >\frac{1}{3}$ is necessary for Corollary 2(2).

**Figure 5:**
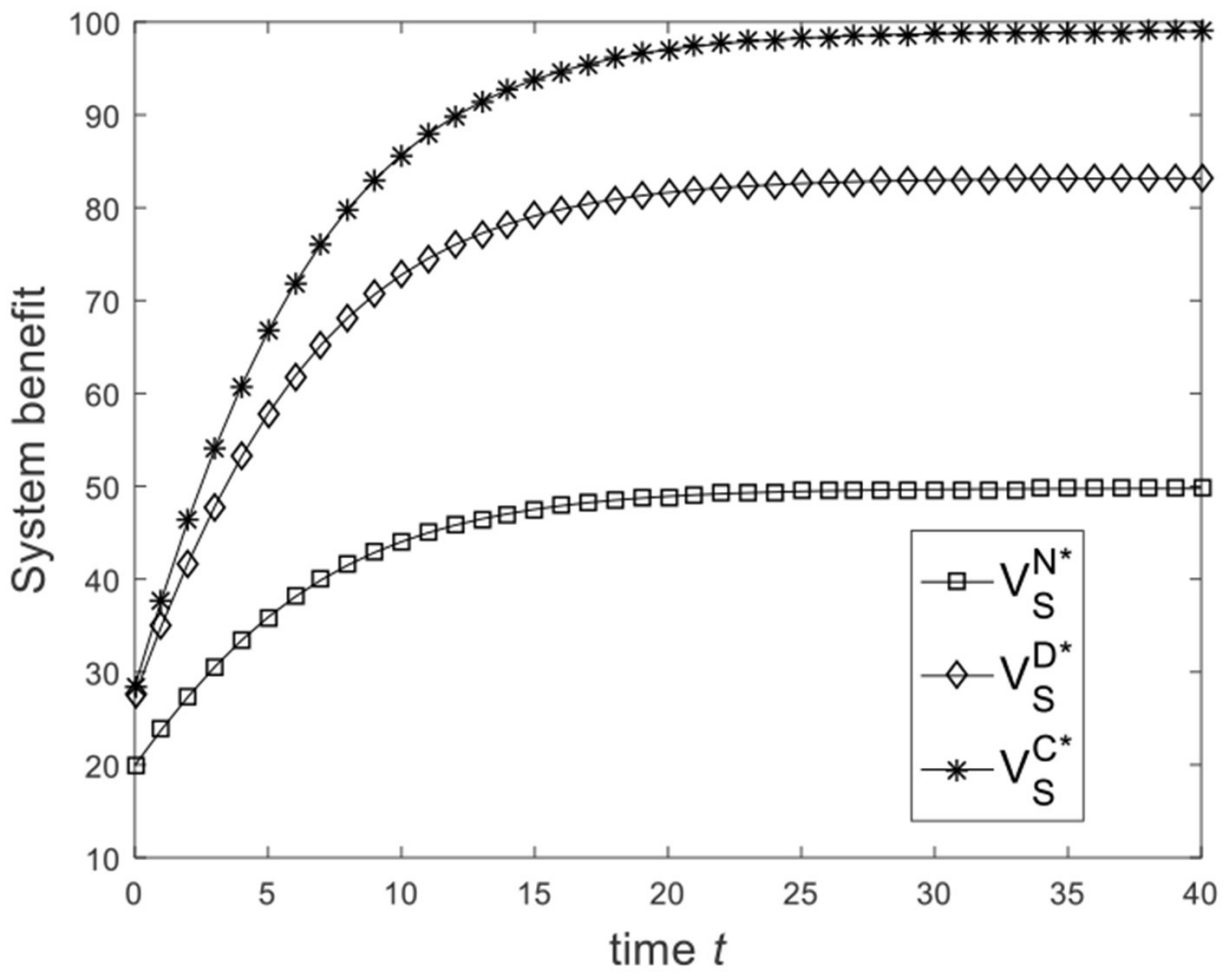
Diagram of the system benefit for $\omega =\frac{2}{3}$.

**Figure 6:**
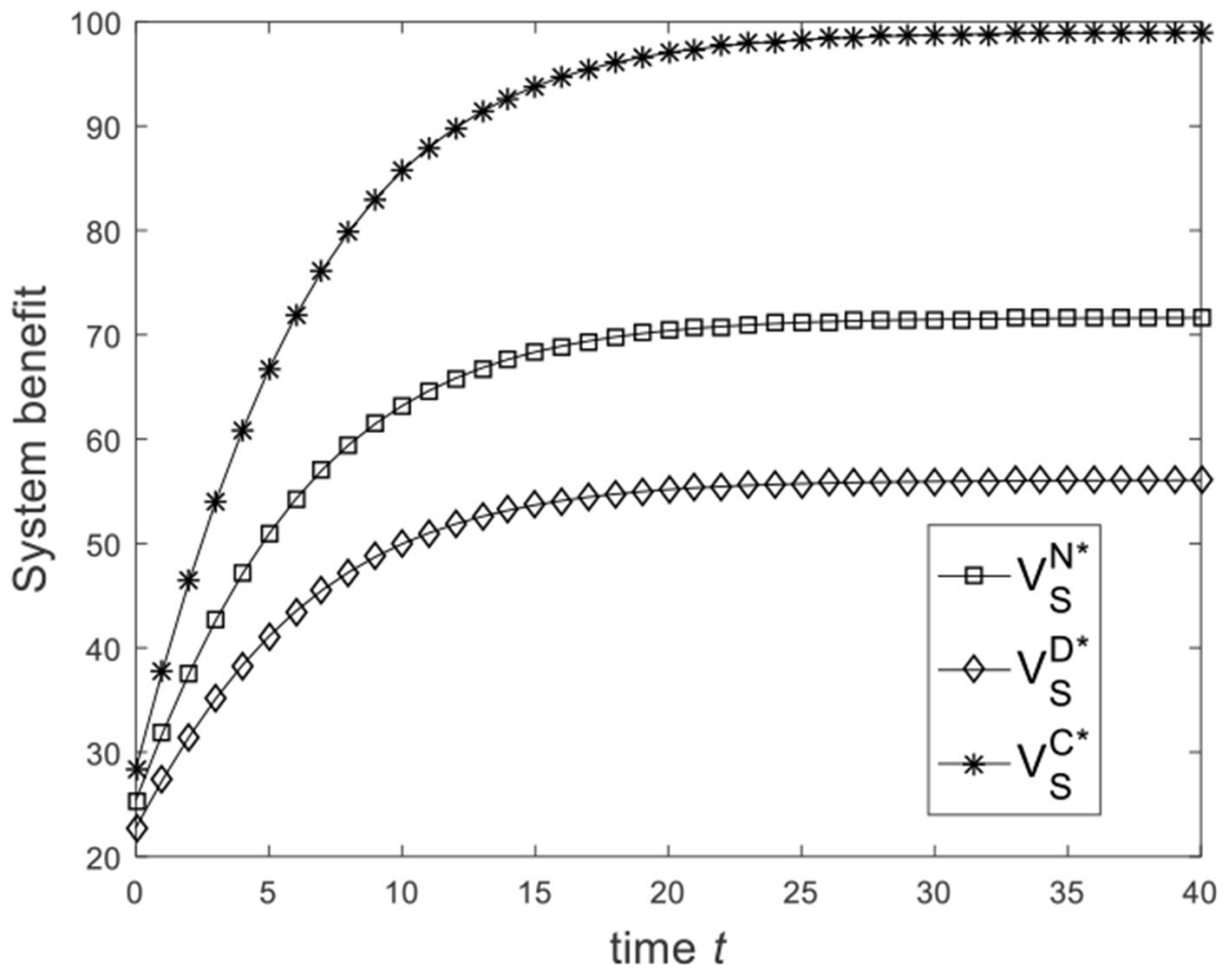
Diagram of the system benefit for $\omega =\frac{1}{6}$.

Let $\omega =\frac{2}{3}$, and the benchmark parameters remain unchanged. Substituting benchmark parameters into (21), (28), and (31), we can get the system benefit under different game scenarios, as shown in Figure 5. From Figure 5, we know that from the Nash non-cooperative game to the Stackelberg master–slave game and then to the cooperative game, the system benefit increases. Figure 5 is consistent with the conclusion of Corollary 4. Let $\omega =\frac{1}{6},$ and the benchmark parameters remain unchanged. Substituting benchmark parameters into (21), (28), and (31), we can get the system benefit under different game scenarios, as shown in Figure 6. From Figure 6, we know that the system benefit in the Stackelberg master–slave game is lower than that in the Nash non-cooperative game. So the condition $\omega >\frac{1}{3}$ is necessary for Corollary 4.

## Conclusion

Considering the simple regulatory system composed of the local government and the enterprise, we establish differential game models to study the regulation of online car-hailing in the era of the sharing economy. We calculate some indicators, such as the optimal regulatory effort of the local government, the optimal regulatory effort of the enterprise, the optimal benefit function of the local government, the optimal benefit function of the enterprise, the optimal benefit function of the system, the optimal trajectory of the service quality level for the enterprise, and the optimal trajectory of the goodwill for the enterprise. Moreover, we compare these indicators under different game scenarios. Combining numerical simulations, we can draw the following important conclusions:

1. In the Stackelberg master–slave game, the optimal ratio of the local government subsidy to the enterprise's regulatory cost is only related to the benefit distribution coefficient ω and has nothing to do with other factors. Furthermore, when $\omega >\frac{1}{3}$, the local government is willing to share the regulatory cost of the enterprise. Otherwise, the local government refuses to share the regulatory cost of the enterprise due to the low regulatory benefit. It can be seen that the benefit distribution coefficient ω is the key for the local government to decide whether to provide a subsidy for the enterprise. When the benefit distribution coefficient $\omega >\frac{1}{3}$, the local government will provide a regulatory cost subsidy for the enterprise, and the service quality level and goodwill of the enterprise will be improved. Therefore, the market of online car-hailing can healthily develop.
2. Compared with the Nash non-cooperative game, in the Stackelberg master–slave game, the optimal regulatory effort of the local government remains unchanged, while the optimal benefit of the local government increases. Furthermore, when $\omega >\frac{1}{3}$, both the optimal regulatory effort and the optimal benefit of the enterprise increase, and Pareto improvement of the benefit for the local government and the enterprise can be achieved. So, compared with the mode that the local government and the enterprise independently carry out regulatory work, the mode that the local government shares the regulatory cost of the enterprise is better. In the real world, the local government can subsidize the enterprise through financial subsidies, tax incentives, and so on, which can achieve a win-win situation for the local government and the enterprise. In fact, sharing the regulatory cost with the local government can motivate the enterprise to increase regulatory input. The enterprise will change from “passive regulation” to “proactive regulation.” Therefore, the enterprise will take the initiative to change and combat the chaos in the online car-hailing market, and the online car-hailing market will healthily develop.
3. Compared with the Stackelberg master–slave game, both the local government and enterprise aim to maximize the system benefit in the cooperative game. So when the benefit distribution coefficient $\omega >\frac{1}{3}$, the optimal regulatory effort of the local government, the optimal regulatory effort of the enterprise, the optimal benefit of the system, the service quality level of the enterprise, and the goodwill of the enterprise all increase. It follows that the system benefit achieves the optimal in a cooperative game. At present, the government's first-line regulation of platforms, drivers, and vehicles will bring an unnecessary burden to the enterprise, which is not conducive to the innovation of new business. The mode of cooperative governance between government and enterprises can reduce the burden of the enterprise, which is conducive to the precise governance of the online car-hailing market. In practice, information sharing and feedback mechanisms should be established as soon as possible, and a new mode of cooperative governance between government and enterprise should be explored. On the one hand, using Internet thinking to innovate the regulatory way, information exchange and resource sharing between government and enterprises can be realized. On the other hand, taking advantage of the technical advantages, talent advantages, and data advantages of online car-hailing platforms, the regulatory efficiency of online car-hailing can be improved. In summary, for the regulation of online car-hailing, the local government and the enterprise should scientifically decide their regulatory effort according to different cooperation modes, their development status, and the market environment. Finally, the local government and the enterprise strive to improve their own benefit or the system benefit.

In this study, it is assumed that the regulatory system is composed of a local government and an enterprise. However, in reality, there is often a complicated relationship between the government and enterprises. Therefore, we further consider the regulatory system composed of one government and multiple enterprises with competitive or cooperative relationships and study the regulation of online car-hailing in the future.

## Data Availability Statement

The original contributions presented in the study are included in the article/supplementary material, further inquiries can be directed to the corresponding author/s.

## Author Contributions

MY was responsible for conceptualization and methodology of the manuscript. YL was responsible for software and investigation of the manuscript. LS and DW were responsible for writing of the manuscript. XL was responsible for supervision, project administration, and validation of the manuscript. All authors contributed to the manuscript and approved the submitted.

## Funding

This study was funded by the National Natural Science Foundation of China (11801352 and 71701122).

## Conflict of Interest

The authors declare that the research was conducted in the absence of any commercial or financial relationships that could be construed as a potential conflict of interest.

## Publisher's Note

All claims expressed in this article are solely those of the authors and do not necessarily represent those of their affiliated organizations, or those of the publisher, the editors and the reviewers. Any product that may be evaluated in this article, or claim that may be made by its manufacturer, is not guaranteed or endorsed by the publisher.
